# Walkability and Greenness Do Not Walk Together: Investigating Associations between Greenness and Walkability in a Large Metropolitan City Context

**DOI:** 10.3390/ijerph18094429

**Published:** 2021-04-21

**Authors:** Faysal Kabir Shuvo, Soumya Mazumdar, S. M. Labib

**Affiliations:** 1 Centre for Urban Transitions, Level 1 EW Building Swinburne University of Technology, Hawthorn, VIC 3122, Australia; 2 South Western Sydney Clinical School, University of New South Wales Medicine, Liverpool, NSW 2170, Australia; Soumya.Mazumdar@health.nsw.gov.au; 3 Population Health Intelligence, Population Health, South Western Sydney Local Health District, Liverpool, NSW 2170, Australia; 4 MRC Epidemiology Unit, Centre for Diet and Activity Research, University of Cambridge, Clifford Allbutt Building, Cambridge CB2 0AH, UK; sml80@medschl.cam.ac.uk

**Keywords:** greenness, green space, walkability, NDVI, Walk Score^®^, sweet spots, spatial modeling, geographical information systems, Australia

## Abstract

Background: The existing environment literature separately emphasizes the importance of neighborhood walkability and greenness in enhancing health and wellbeing. Thus, a desirable neighborhood should ideally be green and walkable at the same time. Yet, limited research exists on the prevalence of such “sweet spot” neighborhoods. We sought to investigate this question in the context of a large metropolitan city (i.e., Sydney) in Australia. Methods: Using suburb level normalized difference vegetative index (NDVI), percentage urban greenspace, Walk Score^®^ (Walk Score, Seattle, WA, USA), and other data, we explored the global and local relationships of neighborhood-level greenness, urban green space (percent park area) with walkability applying both non-spatial and spatial modeling. Results: We found an overall negative relationship between walkability and greenness (measured as NDVI). Most neighborhoods (represented by suburbs) in Sydney are either walkable or green, but not both. Sweet spot neighborhoods that did exist were green but only somewhat walkable. In addition, many neighborhoods were both less green and somewhat walkable. Moreover, we observed a significant positive relationship between percentage park area and walkability. These results indicate walkability and greenness have inverse and, at best, mixed associations in the Sydney metropolitan area. Conclusions: Our analysis indicates an overall negative relationship between greenness and walkability, with significant local variability. With ongoing efforts towards greening Sydney and improving walkability, more neighborhoods may eventually be transformed into becoming greener and more walkable.

## 1. Introduction

Being in natural (or green) environments and walkable neighborhoods are usually the most common types of built environmental recommendations for healthy and sustainable urban living [1,2]. The last decade has observed an upsurge in accumulated evidence on the health and wellbeing benefits of urban green spaces and natural areas [3,4,5]. The evidence base has been generated on multiple reviews that reveal relationships between access to green/natural areas and health outcomes, such as physical health, mental health and wellbeing, mortality, stress reduction and physical activity [6,7]. The pathways linking green exposure with health have also been explored and, studies suggest that the benefits of urban green space operate on human health along multiple interlinking pathways. In this regard, Markevych et al. [8] categorized these pathways broadly into three groups; reducing harms (e.g., mitigating heat via shade provision), restoration (e.g., relief from psychological stressors), and most importantly, instoration (e.g., building health capacities through physical activity).

One of the most underrated types of physical activities is walking, even though it is the most common type of leisure-time or transportation physical activity among all population groups [9,10]. Researchers have found a wide array of health benefits of walking [11,12]. Exposure to greenness may encourage more physical activity, such as walking [13,14], walking in green spaces (“green exercise”) accrue more health benefits, and have additional mental health benefits [10,15]. In principle, a healthy neighborhood design should integrate greenness and walkability as the foundation of the neighborhood’s morphology to promote green walking. While both greenness and walkability are complementary attributes of a neighborhood that promote health and wellbeing, the extent of the complementarity between these two important neighborhood attributes has not been adequately studied [16,17]. Anecdotal common sense knowledge suggests that the most walkable neighborhoods are located in high-density built-up urban neighborhoods with low levels of greenness, and outlying low walkability suburban sprawl is associated with high levels of greenness/green space, hence the term “leafy green suburbia” [17]. Not surprisingly, a small, emerging body of evidence also has been pointing towards an overall negative relationship between greenness and walkability in some major metropolitan neighborhoods in the global north [18,19,20].

To distinguish green spaces, such as parks and playgrounds, from overall greenness, we will henceforward identify them as “green space”. Indeed, this distinction is important to appropriately unpack the relationship between greenness, green spaces and walkability. A common measure of overall greenness is the normalized difference vegetative index (NDVI), which has been shown to be associated with our perception of greenness and better mental health [8,21,22,23]. NDVI considers all photosynthetically active vegetation cover. In contrast, measures based on percent green space may use only designated spaces in land use databases that are labeled as public parks, gardens and other green spaces [6,24]. James, Banay, Hart and Laden [22] note that, while such databases may provide accurate information about designated green spaces, they are not necessarily “green” with, for instance, some parks not containing any vegetation. Thus, while green spaces, such as parks, are important, they may not be particularly relevant if studying opportunities for walking in greenness is the goal.

Considering the aforementioned knowledge gaps and differences in methodological approaches to measuring greenness, our investigation had two salient goals. First, we sought to find the overall relationships of both green space and greenness with walkability in a large metropolitan city (Sydney, Australia). Second, we sought to identify neighborhoods and demarcate discrete zones (or collections of neighborhoods) with similar relationships between greenness and walkability. Thus, for instance, we sought to discover contiguous neighborhoods or discrete zones that were “sweet spots” or neighborhoods with high greenness/walkability and “sour spots” or neighborhoods low on both metrics, borrowing terminology from previous research [19,25]. Identification of the location and extent of these neighborhoods would allow us, (i) to observe the extent to which a large metropolitan city has been able to provide its citizens two key desirable environmental attributes, and (ii) to discuss how neighborhoods that do possess both attributes differ from the neighborhoods that do not.

When investigating global relationships, we adjusted for three confounders. First, we adjusted for area-level socioeconomic status (SES) since high SES neighborhoods are known to be greener and maybe more walkable [26]. In addition, in Sydney, as in many other cities, there is a general trend of suburbs in the city’s core being smaller in size but with greater population density than outlying suburbs. Since these suburbs are also more likely to be walkable and less green, we adjusted for population density and suburb size. We discuss our findings in the context of the local geography of Sydney and the implication of findings for researchers, urban planners, and policymakers.

## 2. Materials and Methods

We used data from the metropolitan area of Sydney, Australia, on walkability and greenness/green space. Our greenness data were NDVI values, while we used publicly available urban green spaces data from the Australian Bureau of Statistics (ABS). We used ordinary linear regression, spatial lag and error models to unpack the global relationship between walkability and greenness/green space. Since Spearman’s rank correlation between NDVI values and percent green space was found to be low (Spearman’s rho = 0.401, t < 0.001), we included both of these variables in the same model. We identified “sweet” and “sour” spots using a multivariate clustering tool. Our data and methods are described in detail below:

### 2.1. Geography of the Study Area

**Figure 1:**
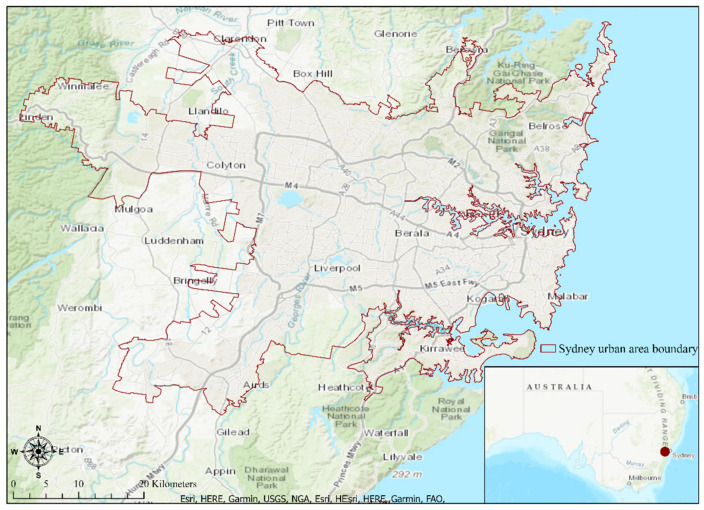
Map of Sydney urban area represented by the urban center and locality boundary (demarcated by the red line).

We have considered the urban centers and locality (UCL) boundary of Sydney (population approx. 5 million) as the boundary of the Sydney metropolitan area [27]. UCLs are defined as collections of the smallest possible geography used by the ABS called Statistical Area 1 s (SA1s), each of which contains between 200 to 800 people ABS [28]. The UCL boundary in Sydney comprises 2009 square kilometers (based on the GIS data obtained from ABS). Figure 1 displays the extent of our study area.

The suburb is the spatial unit of analysis in this study. Suburbs are gazetted localities that are geographically equivalent to urban districts or neighborhoods in the United States of America (USA) or the United Kingdom (UK). Unlike the SA1, which is an artificially constructed geography, suburbs were traditionally informal geographies with large variations in shape and size, making it difficult to define a typical suburb in Australia. The size of the suburbs can vary depending on the character, age, and distance from the city. For example, while the mean size of suburbs in our study area is 3.45 square kilometers, some of the newer suburbs in southwestern Sydney have an average size of 7.16 square kilometers. We generally use the term neighborhood to indicate suburb in our analyses for ease of understanding.

### 2.2. Walkability

Walkability can be measured objectively [29,30] or subjectively [31,32], and we used an objective measure of walkability in this study. The Walk Score^®^ measure, which was developed by a team of scientists in the USA, has been validated and used by urban planning and public health researchers [33,34]. The methodology used by Walk Score^®^ involves several elements [33], including the shortest network distance to several amenities, including educational, retail, recreation, food, recreation and entertainment facilities [35]. The Walk Score^®^ algorithm assigns each location a score between 0 and 100. Further details on Walk Score^®^ are available from the Walk Score^®^ website [36] and from a publication by Duncan, Aldstadt, Whalen, Melly and Gortmaker [35]. Walkability values for our study area were obtained from the Walk Score^®^ website at the suburb/SSC level. We utilized these categorizations in our descriptions. The creators of Walk Score^®^ have suggested the following categorizations for this measure: 90–100 (walker’s paradise: daily errands do not require a car), 70–89 (very walkable: most errands can be accomplished on foot), 50–69 (somewhat walkable: some errands can be accomplished on foot), 25–49 (car-dependent: most errands require a car) and 0–24 (car-dependent: almost all errands require a car.) [36].

### 2.3. Greenness and Green Space Measures

In this study, we utilized NDVI as the greenness metric. NDVI is one of the most commonly used vegetation indices of measuring greenness in the literature [8,21]. We estimated NDVI values for the study area using Sentinel-2 satellite imagery. We processed the NDVI layer from Sentinel-2 satellite images obtained between September 2016 and February 2017. This period was chosen based on the phenological pattern observed in the study area, indicating the growing and maturing season of vegetation after the winter months (June to August). We selected Sentinel-2 images because they have a relatively higher spatial resolution (10 m) compared to the widely used Landsat (30 m) and MODIS (250 m) images. Previous studies indicated that for greenness characterization, Sentinel-2 images might yield higher overall accuracy for detecting vegetation cover in urban contexts compared to relatively low or moderate resolution MODIS or Landsat images [8,37].

We used the Google earth engine to identify images for this period and extracted a composite image, including pixels with the median value for all the pixels identified of this period with cloud cover less than 10%. This processing was conducted to ensure the pixel values represent the seasonality and phenological pattern of the study area, not a single-day snapshot of the vegetation cover. Finally, we used the following standard equation to calculate NDVI;

(1) $$\mathrm{NDVI}=\frac{\mathrm{NIR}-\mathrm{RED}}{\mathrm{NIR}+\mathrm{RED}}$$

where RED refers to the red band (central wavelength 664.6 nm) and NIR is the near-infrared band (central wavelength 832.8 nm) of the Sentinel-2 images [38]. The estimated NDVI values range between −1 and 1, with higher positive values indicating greater green vegetation or greenness [37,39]. We estimated area-level mean NDVI values by using the zonal statistics tool in ArcGIS Pro to extract suburb/SSC level greenness.

In addition to the greenness data, we have also used urban parkland data as an indicator of urban green spaces. Urban parkland data are available at the highest spatial resolution from the ABS, at the mesh block level and updated every 5 years. Mesh blocks comprise approximately 30–60 dwellings and generally do not cross cadastral/parcel boundaries [40]. Mesh blocks that included nature reserves protected and conserved areas, public open space and sporting arena were categorized as green space [40]. Since mesh blocks nest into SSCs/suburbs, using 2016 mesh block-level data, we calculated the proportion of parkland area, termed as a percent of urban green spaces, in the suburbs.

### 2.4. Other Variables

We used the index of relative socioeconomic advantage and disadvantage (IRSAD) score to represent neighborhood-level socioeconomic status for the year 2016. IRSAD scores are published by the ABS, and they are a composite index of area-level socioeconomic status, with higher scoring neighborhoods being more advantaged than lower-scoring neighborhoods. In addition, we also computed population per square kilometer at neighborhoods to represent population density.

### 2.5. Statistical Analyses

We mapped descriptive statistics at the suburb level. To explore the relationships, we utilized three global models: ordinary least squares (OLS), spatial lag model (SLM), spatial error model (SEM), in addition to the spatial multivariate clustering method.

#### 2.5.1. Ordinary Least Squares (OLS)

We used the common form of the OLS model as follows [41]:

Y = a + β_i_X_i_ + ε (2)

where Y is the Walk Score^®^ for each of the neighborhoods (i), a is the intercept, and X_i_ is the set of independent variables and covariables, i.e., NDVI, % of urban green space, population density, IRSAD score. β_i_ is the vector of regression coefficients, and ε is a random error term. The least-squares method estimates β by minimizing the sum of squared prediction errors. The OLS model assumes that the errors are independent and that there is no spatial dependency among them. As such, it also assumes that the observations or independent variables are independent [42]. Spatial dependence of errors may cause underestimation of the variation in the parameter estimates (smaller confidence intervals).

#### 2.5.2. Spatial Statistics

##### Spatial Lag and Error Model

While an OLS model may provide a rough first estimate of the nature of the relationship between green space/greenness and walkability, it is necessary that spatial models be used to obtain appropriate confidence intervals/significance estimates. Thus, we used spatial lag and spatial error models, which are spatial variants of the OLS model, with provisions for spatial dependency adjustment [43].

(a) Spatial Lag Model

In this model, we assumed that Walk Score^®^ in one suburb is affected by the independent variables is not only the suburb itself but also its neighboring suburbs. The spatial lag model is denoted by:

Y = a + β_i_X_i_ + ρW_i_Y + ε (3)

In addition to the parameters in the OLS equation, additional parameters in the spatial lag model equation adjust for spatial dependence. Here, ρ is the spatial lag parameter, and W_i_ is the spatial weights matrix, determined by choice of neighborhood configuration [42]. The weight matrix (W) in this equation indicates the nearness of the suburb at one location that links the outcome variable (Y) to the explanatory variables (X_i_) at that location. Following other researchers [41], we used a Queen contiguity matrix, with a maximum of 16 of the adjacent suburbs, for generating a spatial weight matrix in both the spatial lag and spatial error models.

(b) Spatial Error Model

Unlike a spatial lag model, which assumes the dependent variable autocorrelation to be spatially structured among and across areal units (the suburbs), the spatial error model simply assumes that the error terms have a specific correlation structure:

Y = a + β_i_X_i_ + λW_i_ξ_i_ + ε (4)

In this model, the spatial element of the error term is denoted by ξ_i_, λ specify the correlation between the elements and the ε spatially unrelated error term (as used in OLS).

##### Multivariate Clustering

While the above approaches allow the investigation of the overall global relationship between greenness and walkability, it is possible that local variations exist in this relationship. Specifically, local regions of Sydney may show different patterns of relationships between the two variables relative to other regions. That is to say, some neighborhoods may possess high walkability, high greenness, while others may have low walkability, low greenness or, as described earlier, “sweet and sour spots” [18,25].

**Figure 2:**
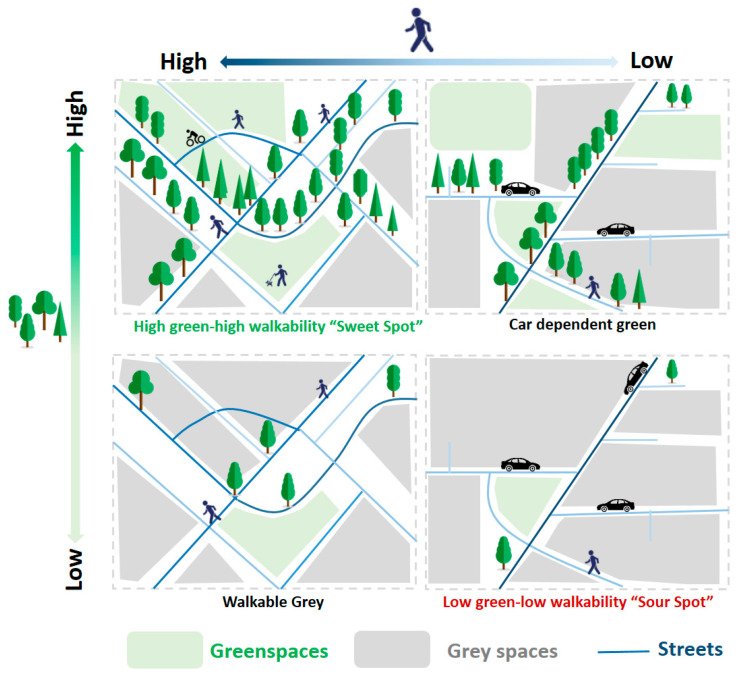
Four-way patterns of the relationship between greenness and walkability. Gray spaces indicate areas that are built up or are otherwise not green.

The concept is illustrated in Figure 2. It is important to note that, while ideally, we expect to identify neighborhoods with the highest (or lowest) possible value of walkability or greenness to able to define sweet and sour spot neighborhoods, this may not be possible in reality, and only neighborhoods with relatively high greenness or walkability may be found. Thus, for instance, a sweet spot may include neighborhoods that are not a walker’s paradise (Walk Score^®:^ 90–100) and instead be very walkable (Walk Score^®:^ 70–89), yet be relatively more walkable than other neighborhoods of Sydney.

To identify neighborhoods with similar relationships between walkability and greenness, we utilize a K Means algorithm, which identifies clusters in multidimensional space with a spatial contiguity constraint. This method ensures feature similarity within clusters while trying to maximize across cluster dissimilarity [43]. While the tool can be used with multiple variables, this analysis was a bivariate analysis using walkability and greenness.

### 2.6. Implementation of the Models

The amount of variation explained by the global models was measured using adjusted R^2^ and Akaike information criteria (AIC). Variance inflation factors (VIFs) were reported for the OLS regression to detect collinearity. All the global models were implemented in GeoDa [44]. Multivariate clustering was implemented in ArcGIS Pro [45].

## 3. Results

**Figure 3:**
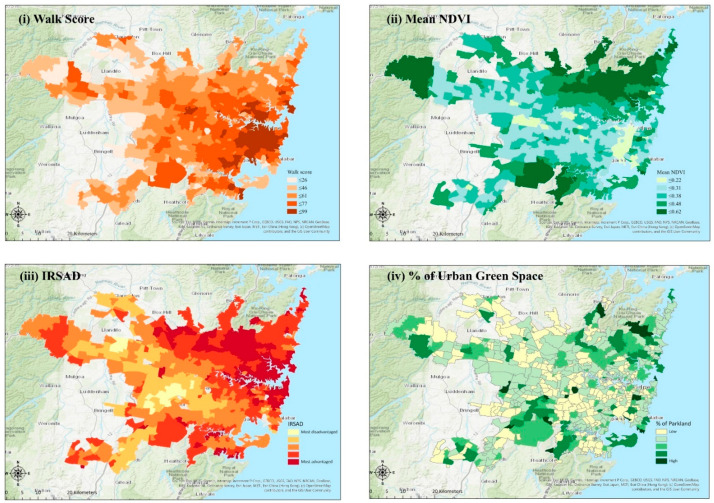
Maps of the main variables examined: (**i**) Walk Score^®^, (**ii**) mean NDVI, (**iii**) IRSAD, (**iv**)% of urban free space.

There are 589 neighborhoods/suburbs within our study area. The variables used in our analyses are summarized in Table 1. Figure 3 provides visualizations of some of the variables. Overall, greenness (NDVI) and percent green space in the study area show large variations indicating that local, geographical analyses are indeed justified. In addition, note the uneven distribution of population density within the Sydney metropolitan area, indicating differing levels and forms of settlement and development. The maps (Figure 2) show that, while green space does not have a specific pattern to its distribution, NDVI values are higher towards the northeastern corner of the map. Other visible patterns are an east-west gradient of IRSAD and a blob of very high walkability in and around the central core of Sydney.

Global OLS model results are presented in Table 2. Walkability, as measured by Walk Score^®^, is negatively associated with greenness measured by NDVI but positively associated with advantage (less disadvantage) and population density. No significant collinearity was found (VIF < 2), and the model explained less than half the variation in walkability. A Moran’s I test of the residual errors showed that errors were highly clustered (Moran’s *I*: 12.3216 and *p* < 0.01), indicating that spatial dependency may exist. The use of spatial lag and error models that accommodate for spatial dependency is, therefore, justified.

Overall, both the spatial lag and error models were significant. The significant negative relationship between Walk Score^®^ and NDVI persisted in the two spatial models, as did the positive relationships with IRSAD, population density and percent green space (Table 3).

**Figure 4:**
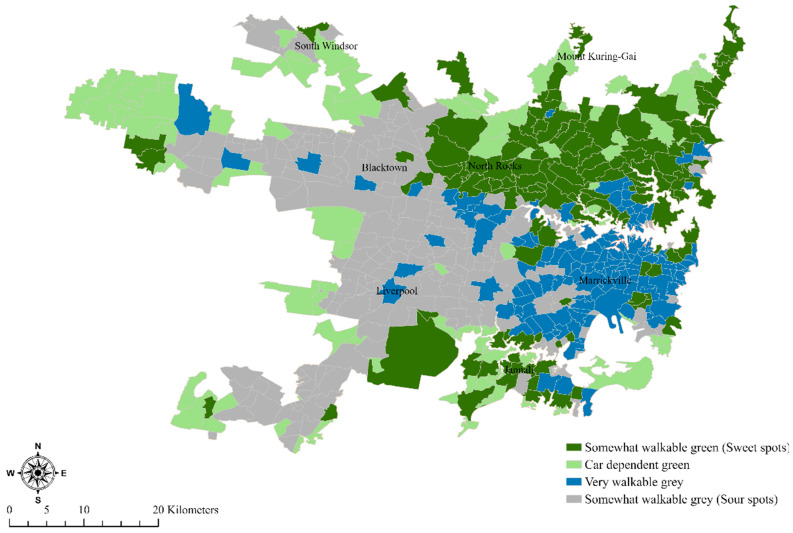
Map of multivariate clusters using Walk Score^®^ and mean NDVI at neighborhoods showing sweet and sour spots.

However, there remains the possibility that, while the global relationship between NDVI and Walk Score^®^ remains negative, there are local variations across the broader regions of Sydney on how the local relationships between NDVI and Walk Score^®^ are distributed. Spatially constrained multivariate clustering allowed us to identify four geographic clusters of neighborhoods across Sydney. The clusters were generally spatially contiguous, with some outliers located outside the general cluster regions. We termed the cluster of neighborhoods as somewhat walkable green (sweet spot neighborhoods), car-dependent green, very walkable gray (with gray implying less green), and somewhat walkable gray (Sour spot neighborhoods) using the Walk Score^®^ terminology described earlier: Table 4 and Figure 4.

The first cluster (sweet spots) in our analysis had a median Walk Score^®^ of 60, which is “somewhat walkable” and a relatively high NDVI of 0.44 (Note that Australian NDVI scores generally range from 0.1 to 0.7 [46]). Sections of the northern beaches, north Shore and some inner northwest neighborhoods of Sydney and a few neighborhoods in the Sutherland Shire to the south of the sections belong to this category. The majority of these sweet spot neighborhoods are sought after and characterized by high property prices [47]. Since this cluster provided the best combination of walkability and greenness, we categorized these neighborhoods as sweet spots. Sandwiched between the two sets of sweet spot neighborhoods in the north and Sutherland Shire lie the inner and central neighborhoods of Sydney with a dense collection of commercial services, offices, and businesses, in addition to high and medium-density residences. Most of this area is part of what we call “very walkable gray”, with a median Walk Score^®^ of 81, with a quarter of neighborhoods having Walk Score^®^ higher than 87. However, residents of these densely built-up neighborhoods must compromise on greenness with a median NDVI of 0.27. Almost all neighborhoods of western Sydney were identified as sour spots, being “somewhat walkable” with a median Walk Score^®^ of 51 and a low NDVI of 0.31. The final group of neighborhoods, which we call “car-dependent green,” consists of neighborhoods, which lie at the edge of, and/or include portions of various national parks and reserves, often lying at the borders of Sydney’s populated/settled neighborhoods, with some very low-density housing. Some neighborhoods are open land either being currently developed or on the course of being developed in the future. These neighborhoods have a median Walk Score^®^ of 30 but a high NDVI of 0.46. There are occasional exclaves of each cluster in other clusters. Thus, for instance, some of the neighborhoods comprised of town centers/central business districts of various western Sydney local governments belong to the “very walkable gray” cluster. This includes the neighborhoods of Liverpool and Bankstown. In addition, sweet spot pockets of “somewhat walkable green” neighborhoods are scattered all over Sydney, including the historic city of Windsor in the northwest and the town center of Camden in southwestern Sydney.

## 4. Discussion

Greenness and walkability are the two critical characteristics of a healthy environment, with the general expectation that the two should co-exist in tandem. Our study shows that, in metropolitan Sydney, the two are significantly negatively associated with each other. Further, local spatial analyses showed that neighborhoods with the highest walkability were not very green, and conversely, neighborhoods with high greenness were car-dependent. Some neighborhoods that had relatively high greenness were only somewhat walkable, and a large number of neighborhoods in western Sydney were not particularly green or walkable. Secondary findings were that there were significant positive associations between green space (parks, reserves, etc.), neighborhood-level advantage, and walkability.

This study has two strengths. First, this study is unique in being one of the first to focus on the specific question of the relationship between greenness and walkability. Other studies have investigated the relationship as part of a larger study. Second, it uses validated walkability metrics (Walk Score^®^), the widely used NDVI, and a unique method to identify local spatial clusters of neighborhoods with similar values of greenness and walkability. The study had several weaknesses. First, while NDVI is a validated metric of greenness [48], recent research suggests that tree canopy cover or Google Street View-based indicators may be better indicators of perceived greenness [23,49]. This is because eye-level greenness visibility showed a better correlation with human perception, and top-down measures of greenness are distinct from eye-level measures [50,51]. Second, the NDVI values in this study varied within a narrow band of between 0.2 and 0.5. This is a drawback stemming from the fact that greenery in Sydney is not particularly “green,” and replicating this study in more tropical locales could provide a greater variation in NDVI values. Third, we used neighborhood-level average values for NDVI, Walk Score^®,^ etc., for our study, which may mask finer variations at smaller scales. This limitation is related to the fallacy of “ecologically-based” based measures of NDVI and Walk Score. Deducing a single value from the aggregation of multivariable characteristics involves ecological fallacies [52,53]. Despite the drawbacks, neighborhoods -level composite indexes were preferred and started to evolve in the UK [54] and USA [55] back in the 1970s to measure health inequality [56]. Therefore, both NDVI and Walk Score^®^ index values available for the population residing within our pre-specified neighborhoods have been used as proxy markers. Fourth, while we have included key confounders in our regression analyses, it is possible that other confounders may have been missed, and the extent to which this would affect our results is not known.

Previous studies from various jurisdictions have noted the negative relationship between greenness and walkability as part of other investigations. Thus, Marquet, Floyd, James, Glanz, Jennings, Jankowska, Kerr and Hipp [20], working on data from San Diego, USA, reported a correlation of around −0.2 to −0.4 between walkability and NDVI greenness. Another Canadian study from British Columbia (BC), investigating the effect of noise and pollution on diabetes, noted a negative correlation of −0.6 between walkability and NDVI [18]. Across the border from BC, some researchers from Seattle noted a similar negative relationship between walkability and NDVI [57]. Finally, a study from Canada exploring the relationship between NDVI, walkability, and air pollution in Canada’s three large metropolises reported negative correlations ranging between −0.5 and −0.6, with high walkability, high greenness postcodes comprising around 3–6% of all postcodes [19]. While all of these studies explore the global relationship between walkability and greenness, our study is unique in exploring the local spatial variation of this relationship. While what drives this relationship merits further investigation, it can be generally understood that neighborhoods with high walkability have not only a high density of roads but also a high density of dwellings and other amenities, such as shopping and transportation. This requires that a significant part of the neighborhood be covered with tarmac or concrete and be otherwise built up. This also means that natural surfaces, grass and nature strips are built over, and backyards/front yards from low-density housing are absent. Thus, unless a deliberate attempt is made to plant trees and shrubbery or plan in greenspace and greenness into the neighborhood, it is likely that high-density growth and increased walkability would lead to a reduction in greenness.

It has long since been known that differentials in demographics, work opportunities, health, and transport opportunities exist between the more and less advantaged neighborhoods of many cities of the global north. The location and distribution of the sweet spots and sour spots in our study expose ongoing issues with environmental justice in these cities. Thus, for instance, in the Canadian study by Doiron, Setton, Shairsingh, Brauer, Hystad, Ross and Brook [19] mentioned earlier, the authors report that deprived neighborhoods were less green and more polluted compared to advantaged neighborhoods. In the case of Sydney, the divide exists between the neighborhoods of eastern and western Sydney, with some commentators calling this the “latte line” divide [58,59]. The latte line is thus an imaginary line dividing eastern and western Sydney, demarcating differences in demographics, health, employment opportunities and socioeconomics. In our analyses, while the sweet spots to the north and the very walkable gray neighborhoods in the inner and central neighborhoods lie to the east of this latte line, most of the sour spots lie west of this line, i.e., to the western Sydney. Indeed, all of Sydney’s ten most expensive neighborhoods (suburbs) lie east of the latte line [60], with half of them being within a sweet spot and the other half having some form of a water view. While our global models adjusted for SES, population density, and suburb density, three variables that differ systematically across the east–west axis, with the eastern suburbs having higher SES, density, and smaller sizes, the negative relationship between greenness and walkability persisted.

While the CBD of Sydney and the many neighborhoods surrounding it provide ample opportunities for utilitarian walking, the otherwise scarce greenness in these neighborhoods poses a problem. Nevertheless, there exists a positive relationship between walkability and percent green space, which implies that people living in highly walkable neighborhoods, such as the very walkable gray areas, may have better access to parks and other public open spaces. This also implies that regions of western Sydney may suffer from a triple whammy of poor walkability, poor greenness and poor green space/parks or public open space. In addition, as mentioned earlier, parks and open spaces are no replacement for high overall greenness since, for instance, better mental health is associated with higher levels of tree canopy [61] and visible greenness [49]. A secondary finding of this research is that a small positive relationship exists between the SES and the walkability of suburbs. This is not surprising, and several studies have reported this before [19,62].

This study points towards the trilemma facing the residents of many metropolitan areas, who must choose between residing in neighborhoods with poor walkability, low green space, high property prices or various combinations of these attributes. However, across the globe, many ongoing processes may introduce change for the better, with, for instance, the World Health Organization releasing a brief on greener cities [63]. In Sydney, many western Sydney local governments are engaging in active tree planting and greenery improvement schemes [64]. Similar efforts to increase accessibility to public transport and walkability in existing built-up neighborhoods are also underway [65]. Some developing neighborhoods in the outer fringes of Sydney, which were categorized as car-dependent green in our analysis, are being planned to accommodate higher residential densities and to support walking in spite of a supposed preference of the Australian for a low-density that is known as a suburbanized lifestyle. Thus, while, from a policy and planning perspective, cities around the world are moving in the right direction, our study shows that urban areas in the developed world may still have a long way to go before their residents are provided the right mix of healthy built urban environments.

We investigated the relationship between two key indicators, walkability and greenness, in this study. Urban planners and geographers often characterize neighborhoods based on multiple indicators, which in addition to these include other indicators, such as access to public transport, crime and employment opportunities. However, exploring cityscapes using multiple indicators, many of which (such as access to public transport and walkability) may be correlated, while useful for planning or house relocation purposes, may be difficult to interpret from a broad-brush geographic perspective. However, such analyses are important and must be implemented, though necessitating a thorough and more intensive, and if necessary qualitative analysis of the cityscape. Such intensive analyses were beyond the scope of this study. In addition, beyond the scope of this study are investigations into optimal combinations of parameters, such as walkability and greenness,—research, which could help inform city planning and management. Other methodological approaches, such as geographically-weighted regression, could also be utilized to investigate the relationship if sufficiently large datasets are available.

## 5. Conclusions

This study shows that urban neighborhoods that are green are not walkable or vice versa. However, there are significant local differences, with some neighborhoods being relatively greener and walkable at the same time compared to other neighborhoods. Our study highlights the planning challenges faced within many urbanized neighborhoods, where the provision of both a high degree of walkability and greenness is a difficult proposition to fulfill, though ongoing efforts may be able to satisfy this requirement to some extent.

## Figures and Tables

**Table 1:** Summary statistics.

Variables	Mean	Min	Max	Std Dev
Neighborhood size (sq. km)	3.45	0.107	60.35	4.29
Walk Score^®^	57.18	0	99	20.12
Mean NDVI	0.35	0.06	0.62	0.10
Percent green space	19.35	0	100	18.30
IRSAD score	1055	623	1207	98.12
Population density (per sq. km)	3363	5	32,681	2990

Abbreviations: sq. km—square kilometers, Std Dev—standard deviation, NDVI—normalized difference vegetative index, IRSAD—index of relative socioeconomic advantage and disadvantage.

**Table 2:** OLS parameter estimates: Association between Walk Score^®^ and mean NDVI after adjusting with other covariables.

Variable	Coefficient (with 95% CI)	VIF	Adjusted-R^2^	AIC
Intercept	28.03 **	---	0.4447	4867.1
Mean NDVI	−81.15 **	1.72		
Percent green space	10.87 *	1.23		
IRSAD score	0.05 **	1.18		
Population density	0.002 **	1.61		
Suburb size (sq. km)	−0.11	1.11		

** Significant at *p* < 0.001; * significant at *p* < 0.05. Abbreviations—sq. km—square kilometers, CI—confidence interval, VIF—variance inflation factor, AIC—Akaike information criterion, IRSAD—index of relative socioeconomic advantage and disadvantage.

**Table 3:** Summary statistics of spatial lag and spatial error models.

Variable	Co-Efficient		Z-Score		R^2^		AICs	
	SLM	SEM	SLM	SEM	SLM	SEM	SLM	SEM
Intercept	14.69	61.19	3.72 **	12.86 **	0.6642	0.6724	4633.01	4645.01
Mean NDVI	−44.60	−71.76	−6.48 **	−8.89 **				
Percent green space	1.92	9.61 × 10^−7^	0.62	5.61 **				
IRSAD score	0.02	0.01	5.52 **	3.56 **				
Population density	0.001	0.001	6.40 **	4.67 **				
Suburb size (sq. km)	0.23	0.47	1.63	3.41 **				
ρ	0.60							
λ		0.71						

** Significant at *p* < 0.001; Abbreviations: sq. km—square kilometers, CI—confidence interval, AIC—Akaike information criterion, SLM—spatial lag model, SEM—spatial error model, NDVI—normalized difference vegetative index, IRSAD—index of relative socioeconomic advantage and disadvantage.

**Table 4:** Variation of median Walk Score^®^ and NDVI across the four clusters. Figures in brackets represent 25th and 75th percentiles, respectively.

Cluster	Median Walk Score^®^	Median NDVI
Somewhat walkable green (sweet spots)	60 (54, 69)	0.44 (0.4, 0.5)
Car dependent green	30 (20, 38)	0.46 (0.41, 0.53)
Very walkable gray	81 (73, 87)	0.27 (0.22, 0.31)
Somewhat walkable gray (sour spots)	51 (45, 58)	0.31 (0.29, 0.35)

Abbreviation: NDVI—normalized difference vegetative index.

## Data Availability

All the data used in this study were gathered through open access sources.
